# Associations between ASA Physical Status and postoperative mortality at 48 h: a contemporary dataset analysis compared to a historical cohort

**DOI:** 10.1186/s13741-016-0054-z

**Published:** 2016-10-20

**Authors:** Thomas J. Hopkins, Karthik Raghunathan, Atilio Barbeito, Mary Cooter, Mark Stafford-Smith, Rebecca Schroeder, Katherine Grichnik, Richard Gilbert, Solomon Aronson

**Affiliations:** 1 Department of Anesthesiology, Duke University Hospital, Durham, NC USA; 2 American Anesthesiology, Mednax National Medical Group, Sunrise, FL USA

**Keywords:** ASA Physical Status, Assessment, Classification, Physical status, Preoperative physical status, Value

## Abstract

**Background:**

In this study, we examined the association between American Society of Anesthesiologists Physical Status (ASA PS) designation and 48-h mortality for both elective and emergent procedures in a large contemporary dataset (patient encounters between 2009 and 2014) and compared this association with data from a landmark study published by Vacanti et al. in 1970.

**Methods:**

Patient history, hospital characteristics, anesthetic approach, surgical procedure, efficiency and quality indicators, and patient outcomes were prospectively collected for 732,704 consecutive patient encounters between January 1, 2009, and December 31, 2014, at 233 anesthetizing locations across 19 facilities in two US states and stored in the Quantum™ Clinical Navigation System (QCNS) database. The outcome (death within 48 h of procedure) was tabulated against ASA PS designations separately for patients with and without “E” status labels. To maintain consistency with the historical cohort from the landmark study performed by Vacanti et al. on adult men at US naval hospitals in 1970, we then created a comparison cohort in the contemporary dataset that consisted of 242,103 adult male patients (with/without E designations) undergoing elective and emergent procedures. Differences in the relationship between ASA PS and 48-h mortality in the historical and contemporary cohorts were assessed for patients undergoing elective and emergent procedures.

**Results:**

As reported nearly five decades ago, we found a significant trend toward increased mortality with increasing ASA PS for patients undergoing both elective and emergent procedures in a large contemporary cohort (*p* < 0.0001). Additionally, the overall mortality rate at 48 h was significantly higher among patients undergoing emergent compared to elective procedures in the large contemporary cohort (1.27 versus 0.03 %, *p* < 0.0001). In the comparative analysis with the historical cohort that focused on adult males, we found the overall 48-h mortality rate was significantly lower among patients undergoing elective procedures in the contemporary cohort (0.05 % now versus 0.24 % in 1970, *p* < 0.0001) but not significantly lower among those undergoing emergent procedures (1.88 % now versus 1.22 % in 1970, *p* < 0.0001).

**Conclusions:**

The association between increasing ASA PS designation (1–5) and mortality within 48 h of surgery is significant for patients undergoing both elective and emergent procedures in a contemporary dataset consisting of over 700,000 patient encounters. Emergency surgery was associated with a higher risk of patient death within 48 h of surgery in this contemporary dataset. These data trends are similar to those observed nearly five decades ago in a landmark study evaluating the association between ASA PS and 48-h surgical mortality on adult men at US naval hospitals. When a comparison cohort was created from the contemporary dataset and compared to this landmark historical cohort, the absolute 48-h mortality rate was significantly lower in the contemporary cohort for elective procedures but not significantly lower for emergency procedures. The underlying implications of these findings remain to be determined.

## Background

The American Society of Anesthesiologists Physical Status (ASA PS) Classification System (CS), introduced in 1941, is routinely assigned to patients prior to procedures where an anesthesia professional is present, and application of this system has become a standard component of anesthetic practice worldwide (Gawande et al. 1999; Saklad 1941). The purpose of the ASA PS CS is to communicate the risk of undergoing any procedure that requires anesthesia, with respect to the patient’s underlying systemic illnesses. The ASA PS scale ranges from 1 through 6 (increasing order of risk) with an additional designation to denote an emergent procedure (“E”), which is defined as a procedure where any further delay would lead to a significant increase in the threat to life or limb (Sankar et al. 2014). Although created as a means of characterizing and easily communicating the extent of systemic illness that is present, application of the ASA PS has evolved as a tool to help inform providers about perioperative procedural risks of morbidity and mortality (Saklad 1941; Owens 2001; Haynes & Lawler 1995).

ASA PS has been shown to correlate with anesthetic morbidity and periprocedural outcomes in several surgical case selective studies, but the association between ASA PS and death within 48 h of surgery has not been quantified in a large procedural non-selective dataset in nearly five decades (Rauh & Krackow 2004; Marcario et al. 1997; Sauvanet et al. 2005). Though too blunt a tool to be used for casemix adjustment, ASA PS designations have further dimensions in today’s era of “value-based” reimbursement models where risk adjustment is necessary prior to comparisons of perioperative rates of morbidity, mortality, and utilization of healthcare services across facilities and health systems. Increasingly, providers and payers rely on risk-adjusted benchmarks to evaluate the value of care being delivered, while hospitals and providers are being held increasingly accountable for adverse outcomes (Hirsch et al. 2015; Bjorgul et al. 2010; Skaga et al. 2007; Han et al. 2004). Therefore, we tested the hypothesis that there is an association between ASA PS classification and 48-h periprocedural mortality in a large contemporary cohort, using prospectively collected and validated data. In addition, we evaluated how this association may have changed over time by comparing these findings with data from a landmark historical publication that compared the same system of classification with the same outcome (48-h mortality).

## Methods

Following Duke University Health System Institutional Review Board (IRB) approval, data over a recent 6-year period (January 1, 2009, to December 31, 2014) were procured from the quality assurance (QA) database of a large community-based anesthesiology group practice (American Anesthesiology Inc.). During this study period, this group practice provided anesthesia services for over 700,000 elective and emergency patient encounters across 233 inpatient and outpatient locations at sites without physician-anesthesiologist trainees. Hospitals ranged in bed sizes (fewer than 25 beds to more than 200 beds), settings (urban/rural), and payer mix. The anesthesia group utilizes an internally designed quality improvement program, the Quantum™ Clinical Navigation System (QCNS), across its entire system. Every patient receiving an anesthetic during the study period had a QA sheet filled out by the anesthesiology care team.

In addition to demographic and clinical information at the patient level, information on the anesthetizing location, type of providers involved, anesthetic and analgesic techniques, and performance and efficiency indicators (such as case cancelation, delay in case starts) were collected prospectively, along with several important clinical outcomes within the 48-h period following completion of the procedure. Members of the care team (anesthesiologists, nurse anesthetists, PACU RNs) entered data into the QA sheet, which was independently validated by a QA nurse that completes the sheet within 48 h of the patient’s care continuum. Data were verified for accuracy and completeness by dedicated quality control nurses at each site and electronically transferred to a centralized database. The QCNS system collects comorbidities, anesthetic and procedural information, and outcomes in a homogeneous fashion using a standard set of elements across the entire set of participating sites for every case. Hence, problems such as a lack of independent validation of QA sheet data, variable acquisition of data across different information technology platforms, and heterogeneous data definitions are minimized. Data were collected and analyzed from consecutive QA forms for anesthetics delivered to patients between January 2009 and December 2014 at all clinical sites. Patients who did not have an ASA status recorded, or who had an ASA PS assignment of 6, were excluded from the analysis.

### Statistics

Incident death within 48 h of procedure was cross-tabulated by ASA PS status separately for elective and emergent procedures. Overall mortality rates in the elective and emergent cases were compared using a two-sided exact binomial test. Utilizing the ordinal properties of ASA PS, Cochran-Armitage trend tests were used to evaluate the association between increasing ASA PS (in five categories) and mortality.

In order to compare the association between ASA PS and 48-h mortality in the QCNS dataset to the historical cohort (adult males at US naval hospitals), we extracted the analogous mortality data from the publication by Vacanti in 1970 and created a comparison cohort of adult males in the QCNS dataset. Differences in 48-h mortality between the two cohorts were compared by elective and emergent status via exact binomial tests and within ASA PS using exact chi-square tests followed by the Hochberg multiple comparison correction. All data extraction and analyses were performed with SAS software, version 9.4 (SAS Institute Inc., Cary, NC) or R version 3.1.1, and significance was assessed at the 0.05 level.

## Results

The initial QCNS dataset consisted of 732,704 unique patient encounters, of which 728,902 had ASA PS between 1 and 5. There were 3676 patient encounters with missing ASA status and 126 with an ASA PS of 6.

**Fig. 1:**
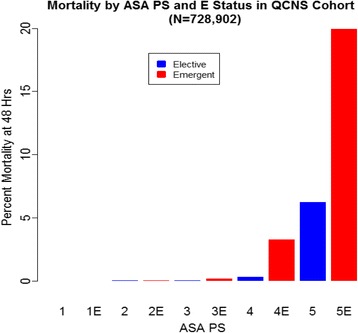
Mortality in full QCNS cohort by ASA PS (*N* = 728,902)

We found a significant trend toward increased 48-h mortality with increasing ASA PS classification for patients undergoing both elective and emergent procedures (*p* < 0.0001). Additionally, 48-h mortality was significantly higher among patients undergoing emergent rather than elective procedures (1.27 versus 0.03 %, *p* < 0.0001). Of the 581 patients that died, 34.6 % had an ASA PS designation of “5” (*n* = 22) or “5E” (*n* = 179). Of those patients with an ASA PS designation of 5 (or 5E), 16 % died within 48 h of surgery (Table 1 and Fig. 1).

**Table 1:** Full QCNS cohort. Given 732,704 unique encounters in the QNCS database, 728,902 encounters had recorded ASA

ASA PS E status	Patients		Deaths	
	*N*	% of cohort	*N*	% of ASA PS E status
1	92,227	12.653	1	0.001
2	367,161	50.372	9	0.002
3	195,829	26.866	54	0.028
4	45,118	6.190	137	0.304
5	353	0.048	22	6.232
1E	3018	0.414	0	0.000
2E	12,188	1.672	4	0.033
3E	7109	0.975	11	0.155
4E	5000	0.686	164	3.280
5E	899	0.123	179	19.911

There were four deaths among the 3676 patients with unknown ASA status, mortality rate of 0.11 % in that group

**Fig. 2:**
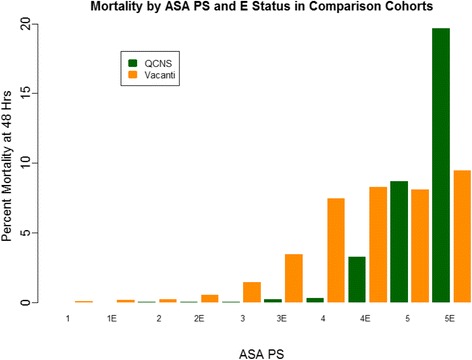
Mortality in QCNS comparison cohort and published Vacanti cohort by ASA PS

In the historical cohort from the Vacanti study, adult male patients were admitted to 11 US naval hospitals between July 1964 and June 1966 (Vacanti et al. 1970). To compare our cohort to this group, we used an “adult male” restriction to the entire QCNS dataset. Using 732,704 unique encounters captured and stored in the QCNS database, we identified 638,626 adults (>17 years of age), of whom 243,500 were males (33.2 %). Of these 243,500 adult males, 1397 (0.06 %) were excluded from the analysis because an ASA PS designation was not recorded (*n* = 1,331), or an ASA status of 6 was designated (*n* = 66). In our contemporary cohort, we found that 306 patients died within 48 h. Of these 306 deaths, 117 occurred among the 232,065 patients that underwent elective surgeries (48-h mortality rate 0.05 %) versus 189 that occurred among the 10,038 patients that underwent emergent procedures (48-h mortality rate 1.88 %). As in the full QCNS cohort, the 48-h mortality rate in the contemporary adult male cohort was significantly higher for emergent patient encounters than elective patient encounters (*p* < 0.0001), and we observed statistically significant trends toward increasing 48-h mortality with higher ASA PS strata in both the elective (*p* < 0.0001) and emergent (*p* < 0.0001) patient cohorts (Table 2 and Fig. 2).

**Table 2:** Comparison QCNS cohort; 242,103 encounters were for adult males that had recorded ASA 1–5

	Patient distribution				48-h mortality				
ASA PS E status	QCNS		Vacanti		QCNS		Vacanti		*p* value
	*N*	%	*N*	%	*N*	%	*N*	%	
1	23,071	9.529	43,964	64.286	0	0.000	32	0.073	<0.001
2	104,997	43.369	10,626	15.538	2	0.002	24	0.226	<0.001
3	80,649	33.312	2928	4.281	28	0.035	42	1.434	<0.001
4	23,198	9.582	523	0.765	74	0.319	39	7.457	<0.001
5	150	0.062	37	0.054	13	8.667	3	8.108	1.000
1E	820	0.339	6739	9.854	0	0.000	11	0.163	0.782
2E	3223	1.331	1975	2.888	1	0.031	10	0.506	0.001
3E	2850	1.177	698	1.021	6	0.211	24	3.438	<0.001
4E	2662	1.100	327	0.478	87	3.268	27	8.257	<0.001
5E	483	0.200	571	0.835	95	19.669	54	9.457	<0.001

The published Vacanti study included 68,388 adult navy hospital patients. Hochberg multiple comparison corrected exact chi-square test *p* values comparing mortality rate within the same ASA PS E status group between the two cohorts

Using data from the Vacanti paper published in 1970, we found a significant trend toward increasing 48-h mortality with increasing ASA PS (*p* < 0.0001) and an overall 48-h mortality rate among patients undergoing elective procedures that was significantly lower than that among patients undergoing emergent procedures (0.24 versus 1.2 %, *p* < 0.0001). While the overall absolute risk of death among patients undergoing elective procedures is significantly lower in the contemporary cohort than that in the historical cohort (0.05 % in the QCNS dataset versus 0.24 % in the Vacanti study), there is not a significant decrease in 48-h mortality for patients undergoing emergent procedures (1.88 % in the QCNS dataset versus 1.2 % in the Vacanti study (Table 2 and Fig. 2)).

## Discussion

In this study, we showed that there was a statistically significant association between increasing ASA PS class and increased risk of 48-h mortality following an anesthetic in a large contemporary cohort of over 700,000 consecutive anesthetics delivered across over 200 locations, using prospectively collected and validated data. Furthermore, our subgroup analysis on adult men suggests that the risk of death within 48 h of an anesthetic increased with increasing ASA PS designations as well. This trend was observed for patients undergoing both elective and emergent procedures. However, the risk of 48-h mortality following an anesthetic was significantly lower in the contemporary cohort when compared with that in the historical cohort for patients undergoing elective procedures. This association was not observed for patients undergoing emergent procedures. Interestingly, the 48-h risk of mortality for patients undergoing emergent procedures was lower in every ASA PS stratum from 1E through 4E when compared to the historical cohort, but the mortality rate in the ASA PS 5E stratum (moribund patients) was higher in the contemporary cohort (19.7 versus 9.5 % in the historic cohort). This large difference in outcomes among ASA PS 5E patients may explain the increase in overall 48-h mortality following an anesthetic that was observed for patients undergoing emergent surgery in the contemporary cohort.

Our findings are generally consistent with a recent report from the National Anesthesia Clinical Outcomes Registry (NACOR) (Nunnally et al. 2015). However, unlike the NACOR data files that are heterogeneous in content by design (i.e., ranging from a minimal dataset to full reporting from the electronic anesthesia information management system) and have variable authentication processes (i.e., through a “variety of automated routines” and through periodic “outlier” reviews by “Anesthesia Quality Institute personnel”), each of our quality assurance forms was individually completed in a consistent manner across all clinical sites and validated by nurses assigned with the specific task of ensuring QA data assurance (Nunnally et al. 2015). ASA PS scores have been correlated with other outcomes (such as operating times, hospital length of stay) as well as postoperative resource utilization (Daabiss 2011; Ridgeway et al. 2005; Tang et al. 2001). Our study provides additional value to this body of literature by providing insight on current practice, as viewed within a historical context; the reductions in the absolute risk of 48-h mortality following an anesthetic that we observed could be expected when considering advances in population health, medical, anesthetic, and surgical care over the past several decades. Finally, the observed increase in mortality among moribund patients (ASA PS 5E) in recent years is noteworthy. Compared with the 1960s when 1 in 12 patients with an ASA PS 5E designation died within 48 h of an anesthetic, our study found that 1 in 5 such patients will die within 48 h of anesthetic. This finding might imply an increase in the incidence of surgical and other procedural interventions in a group of patients that might not have been surgical candidates only decades ago.

There are a few key limitations to this study. Potential underlying differences in the procedure and patient mix between these two cohorts confound further meaningful analyses of the association between ASA PS and 48-h mortality across different time periods. Hence, the exact reason for this difference can only be speculated. These findings could be related to one or more uncontrolled and potentially confounding variables. Surgical procedure-specific characteristics may have changed over time, making the procedure itself safer. In addition, anesthesia-specific risks may have changed over time as several new monitoring technologies are available and the pharmacokinetics of newer induction and maintenance agents may portend safer anesthesia options than those used in the 1960s. It is also reasonable to assume that patient-specific characteristics may be different today than they were in the 1960s; chronic disease management has improved with enhanced workflows, patient record management, and medications. These changes may actually make patients more optimized for surgery, despite having a higher disease burden. It is also possible that the documentation pattern of anesthesiologists has changed over time. Furthermore, as is the case with any large, retrospective database analysis, it is possible that data collected at the point of care may not accurately reflect patient characteristics or patient outcome. We believe this to be highly unlikely in the QCNS dataset as patient death within 48 h of the procedure is an event that every practitioner filling out the QA form is likely to define similarly and capture accurately within the QCNS system. Furthermore, the QA process for the Quantum database is robust. It is important to note that the data used from the contemporary database is for consecutive anesthetics, *not* consecutive patients. Thus, it is possible that repeat patients could have skewed the results of the study. The size and nature of the dataset makes this condition unlikely to significantly impact our results.

## Conclusions

This study shows that data from a large, contemporary, multi-institutional, cohort reaffirm the association between increasing ASA PS designation and increased mortality within 48 h of an anesthetic for both elective and emergent procedures was initially established more than five decades ago. While the associated trends in the historical cohort and the contemporary cohort were similar, a direct comparison of the two datasets suggested that the risk of mortality within 48 h of an anesthetic may be decreased today for all elective procedures and emergency procedures when the ASA PS is between 2E and 4E (no statistically significant change for 1E). These findings could be attributed to advances in population health as well as improvements in medical, anesthetic, and surgical care over the past several decades. The observed increase in risk of 48-h mortality in 5E patients may indicate a change in practice whereby surgery is offered today for extremely high-risk patients, when it may not have been offered only five decades ago. This latter observation is especially relevant because understanding perioperative mortality risk can significantly impact management of risk tolerance in a value-based medicine environment that focuses on improving the cost effectiveness of care for the population.

## Abbreviations

ASA: American Society of Anesthesiologists
CS: Classification System
IRB: Institutional Review Board
NACOR: National Anesthesia Clinical Outcomes Registry
PS: Physical status
QA: Quality assurance
QCNS: Quantum Clinical Navigation System
